# Investigations of Pro- and Anti-Apoptotic Factors Affecting African Swine Fever Virus Replication and Pathogenesis

**DOI:** 10.3390/v9090241

**Published:** 2017-08-25

**Authors:** Linda K. Dixon, Pedro J. Sánchez-Cordón, Inmaculada Galindo, Covadonga Alonso

**Affiliations:** 1 The Pirbright Institute, Ash Road, Pirbright, Woking, Surrey GU24 0NF, UK; pedro.sanchez-crdon@apha.gsi.gov.uk; 2 Department of Biotechnology, Instituto Nacional de Investigación y Tecnología Agraria y Alimentaria, INIA, Ctra. de la Coruña Km 7.5, 28040 Madrid, Spain; galindo@inia.es (I.G.); calonso@inia.es (C.A.)

**Keywords:** African swine fever virus, apoptosis, A179L, A224L pathogenesis

## Abstract

African swine fever virus (ASFV) is a large DNA virus that replicates predominantly in the cell cytoplasm and is the only member of the *Asfarviridae* family. The virus causes an acute haemorrhagic fever, African swine fever (ASF), in domestic pigs and wild boar resulting in the death of most infected animals. Apoptosis is induced at an early stage during virus entry or uncoating. However, ASFV encodes anti-apoptotic proteins which facilitate production of progeny virions. These anti-apoptotic proteins include A179L, a Bcl-2 family member; A224L, an inhibitor of apoptosis proteins (IAP) family member; EP153R a C-type lectin; and DP71L. The latter acts by inhibiting activation of the stress activated pro-apoptotic pathways pro-apoptotic pathways. The mechanisms by which these proteins act is summarised. ASF disease is characterised by massive apoptosis of uninfected lymphocytes which reduces the effectiveness of the immune response, contributing to virus pathogenesis. Mechanisms by which this apoptosis is induced are discussed.

## 1. Introduction to African Swine Fever Virus

African swine fever virus (ASFV) is a large double-stranded DNA virus that replicates predominantly in the cell cytoplasm. The virus causes an acute haemorrhagic fever, African swine fever (ASF), in domestic pigs and wild boar with lethality approaching 100%. In its long-term reservoir hosts in East Africa, warthogs, bushpigs and soft ticks of the *Ornithodoros* species, ASFV causes long-term persistent infections without significant clinical signs.

The disease has a high socio-economic impact upon affected countries in sub-Saharan Africa, Sardinia, Russia and Eastern Europe. Since it spread to Georgia in 2007, ASF has extended through the Trans-Caucasus, Russian Federation and Eastern Europe including EU countries in the Baltic States and Poland [1,2,3]. Most recently, ASF spread in June 2017 to the Czech Republic and in August to Romania [4].

ASFV is the only member of the *Asfarviridae* family. Several large DNA viruses that infect amoeba, including Faustovirus, Kaumoebavirus and Pacmanvirus, are distantly related to *ASFV* and share about 30 conserved genes. These have genomes of approximately 400 kbp, considerably larger in comparison to the ASFV genome of 170 to 193 kbp [5,6,7].

The ASFV genome encodes many non-essential proteins that have important roles in evading host defences. These include proteins that inhibit type I interferon responses, the main early innate antiviral response, and proteins that inhibit apoptosis. The target cells for ASFV replication are mononuclear phagocyte system cells with key roles in activation of innate and adaptive responses. Manipulation of the function of these cells can profoundly affect the host’s response to infection.

In this review, we describe different impacts of ASFV infection on apoptosis. These include the inhibition of apoptosis in infected cells to facilitate virus replication. We also review the massive induction of apoptosis in uninfected cells, particularly lymphocytes, which is a characteristic of acute ASF disease [8].

## 2. Induction of Apoptosis in Infected Cells

**Figure 1:**
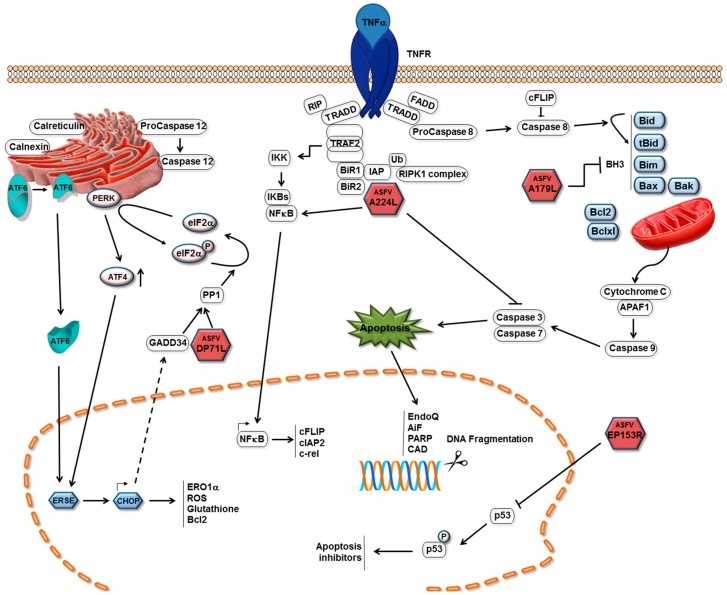
Mechanisms of apoptosis inhibition by African swine fever virus (ASFV). Pathways by which ASFV inhibits induction of apoptosis in infected cells and ASFV proteins are shown as red hexagons with the name of the protein inside. The ASFV A179L Bcl-2 family protein binds to and inhibits several BH3 only domain pro-apoptotic proteins. The A224L IAP-family protein binds to and inhibits caspase 3 and activates nuclear factor kappa-light-chain-enhancer of activated B cells (NF-κB) signalling, thus increasing expression of anti-apoptotic genes including *cFLIP*, *cIAP2* and *c-rel*. The DP71L protein recruits protein phosphatase 1 to dephosphorylate eIF2a, restoring global protein synthesis and inhibiting transcriptional activation of pro-apoptotic CCAAT-enhancer-binding protein homologous protein (CHOP). The EP153R protein inhibits activation of the p53 protein.

The induction of apoptosis in infected cells is an important mechanism by which host cells restrict virus replication. Activation of this process can prevent viruses from completing their replication cycle and thus reduce production of infectious progeny viruses. In common with other viruses, ASFV infection of cells was shown to induce apoptosis, as it induces caspase 3 activation (Figure 1). Other caspases that are activated previous to execution caspase 3 are caspase 9, which is characteristic of the mitochondrial pathway of apoptosis and caspase 12, which is associated with endoplasmic reticulum (ER) stress [9]. In fact, caspase 3 activation after infection occurs in the absence of virus protein synthesis or DNA replication [10]. Inhibition of endosomal acidification blocked the induction of apoptosis as did UV-inactivation of virions. These results suggested that a step including fusion of the viral membrane with the endosomal membrane or virus uncoating, could be involved in initial induction of apoptosis following ASFV infection [10]. Perturbation of membranes as a consequence of fusion or disruption can initiate signalling pathways that lead to cell death [11,12]. Another mechanism for induction of apoptosis involves the interaction of the ASFV structural protein E183L/p54 with the light chain of dynein (DLC8), the microtubule motor protein [13]. The binding site of E183L/p54 to DLC8 dynein is similar to that by which the pro-apoptotic Bcl-2 family member Bim-3 binds. It was suggested that E183L/p54 induces apoptosis by displacement of Bim-3 from microtubules [13,14]. The latter would account for apoptosis induction by the mitochondrial pathway, however, recent evidence has shown that ER stress plays an important role in apoptosis induction after ASFV infection [9]. ER stress might be elicited by the large amounts of viral proteins that are synthesized and accumulate in infected cells potentially overloading the ER protein folding capacity. ER chaperones calnexin and calreticulin are markedly increased 16 hours post-infection (hpi) and protein disulfide isomerase (PDI) at later infection time points (48 hpi). Also, there is a marked increase in caspase 12 activation which is characteristic of ER stress and induces apoptosis. This apoptosis induction might be beneficial for viral spread. In fact, there is a marked activation of ATF6 which was translocated to the nucleus to activate transcription of chaperone-encoding genes and ATF4 only at 48 hpi. It was reported that inhibition of ATF6 action results both in inhibition of all caspases activation and viral production [9].

Inhibition of apoptosis will favour virus replication during the process of progeny virion production. Conversely, at the later stages of infection, it may be advantageous for viruses to induce apoptosis. This would facilitate virus spread by increasing virus release from the cell but avoiding the induction of inflammatory signals that could activate an immune response to clear infection. Apoptosis and the presence of ASFV particles in apoptotic bodies have been observed at late stages of infection [15]. Uptake of these apoptotic bodies into macrophages mediated by phosphatidyl serine receptors could be another route for infection. Induction of apoptosis has been observed in cell culture in ASFV-infected macrophages and in vascular endothelial cells [16].

## 3. Inhibiting Apoptosis in the Infected Cell

In the early 1990s *ASFV* genes with similarities to known apoptosis inhibitory families were identified. These included one protein with similarity to Bcl-2 family members and one with similarity to IAP family members [17,18,19]. These were proposed to inhibit the induction of apoptosis in infected cells, thus promoting cell survival and favouring virus replication. Subsequently, additional ASFV proteins have been shown to regulate cell death pathways (see Figure 1).

### 3.1. Inhibitors of Apoptosis in ASFV-Infected Cells

#### The ASFV Bcl-2 Family Member A179L

Early studies focused on defining the functions of the predicted ASFV-encoded apoptosis inhibitors, including a Bcl-2 family member. The Bcl-2 family proteins contain up to four Bcl-2 homology regions (BH1-4) that are key to their functions as either anti- or pro-apoptotic. The apoptosis inducers include BH3-only proteins which sense cellular damage and initiate the death process and the Bax and Bak proteins that act downstream of BH3-only proteins to permeabilise the mitochondrial outer membrane [20,21]. Bax is primarily cytosolic, translocating to the mitochondrial outer membrane (MOM) after an apoptotic stimulus. Bax and Bak activation is induced by the expression of BH3-only proteins [22]. The BH3-only proteins include Bim, Bid, Puma, Noxa, Bmf, Bik, Bad and Hrk, and function either by directly activating Bak and Bax, or sequestering and neutralizing the pro-survival Bcl-2 members. BH3-only proteins engage the canonical ligand-binding groove on the pro-survival proteins as an α-helix [23].

**Figure 2:**
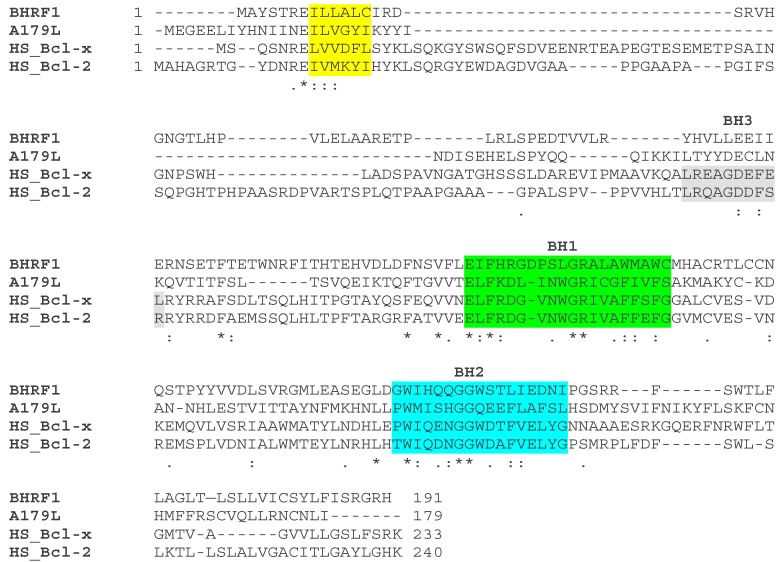
Sequence comparison of ASFV A179L protein with other Bcl-2 family proteins. The ASFV A179L (Interpro annotation P42485) protein sequence was compared with those from EBV BHRF1 (P03182), human Bcl-xL (Q07817) and Bcl-2 (P10415). BH domains are shown as coloured backgrounds. The BH4 domain is shown in yellow, BH3 in grey, BH1 in green and BH2 in turquoise. Amino acid identities between the sequences are shown as asterisks * and similarities as double (:) or single (.) dots. Amended from [30] http://jvi.asm.org/content/91/6/e02228-16.full?sid=d5398579-2b8b-47c3-8fbc-c68ac6dc3ddf.

The A179L protein sequence contains domains similar to all BH domains including a well conserved BH3 domain in the centre [24]. The A179L protein is very well conserved in different ASFV isolates which share between 94% and 99% amino acid identity across 179 amino acids encoded by the entire protein. The similarity with cellular Bcl-2 proteins varies between 33% in mouse and humans, 34% in bovines and 30% in zebrafish. The sequence is most conserved with cellular Bcl2-family proteins between residues 70 to 138 which contain the BH1 and BH2 predicted domains, however A179L lacks the transmembrane domain of cellular Bcl-2 (see Figure 2). The A179L protein was shown to be expressed at both early and late times post-infection in macrophages as an 18 kDa protein [17]. In later studies, A179L was shown to localise at the mitochondria or endoplasmic reticulum [25]. This protein suppressed apoptotic cell death in different cellular systems. For example, it suppressed the strong apoptosis induced by the double-stranded RNA-activated protein kinase (p68) in HeLa and BSC-40 cells [26] and the apoptosis induced by inhibitors of macromolecular synthesis in the human myeloid leukemia cell line K562 [27], demonstrating it was a functional member of the Bcl-2 family. Expression of A179L in insect cells from recombinant baculovirus extended the survival time of infected insect cells when grown as a monolayer but not in suspension suggesting the anti-apoptotic activity may be cell-anchorage dependent [28]. This observation has not been followed up using mammalian cells or porcine macrophages but may be relevant to the understanding of ASFV replication in vivo.

Using a yeast two-hybrid assay, A179L was shown to bind to several BH-3 only proteins, including the activated truncated forms of the Bid protein. Co-precipitation of A179L with active truncated Bid-(p13 and p15) and binding of A179L to other BH-3 domain proteins and pro-apoptotic Bak and Bax (Figure 1) was observed in pull-down assays but not to full length Bid and Noxa [29]. In addition to its role in apoptosis, A179L modulates autophagy via interaction with Beclin-1, and inhibits autophagosome formation under starvation conditions [25]. This was further investigated by determining the kinetics of binding of BH-3 motif peptides to A179L [30]. The results confirmed high affinity binding with several pro-apoptotic BH-3 motifs including Bid, Bim, Puma and also with Bak and Bax. Lower affinity binding was detected to Bmf, Bik and Bad. Hrk and Noxa bound with much lower affinities [30]. The crystal structure of A179L bound to Bid and Bax BH-3 motifs was determined to understand the basis for the unusual promiscuity of A179L. The configuration of the A179L ligand binding groove and some specific interactions suggested a mechanism for the broad specificity. The region corresponding to the α3 helix in Bcl-2 proteins that forms one side of the A179L ligand-binding groove is not helical, and adapts an extended configuration. Elevation of B-factors in this region suggested considerably flexibility in binding, as would be required to engage such a broad range of pro-death Bcl-2 ligands [30]. However, further studies are required to investigate the possible influence of other homology regions in binding specificity. As yet, there are few studies on the role of A179L protein in virus infection and the importance of its interaction with different binding partners. It is possible that the promiscuity in A179L binding is required for its function in both arthropod and mammalian cells.

### 3.2. The ASFV IAP-Family Member

#### 3.2.1. Roles of Cellular IAPs

The IAP inhibitor of the apoptosis protein family were first identified in baculovirus and shown to inhibit cell death in insect cells [31]. Recently, the baculovirus inhibitor of apoptosis Op-IAP3 was shown to bind and stabilise an insect cellular IAP, preventing the virus-induced degradation of this protein. Formation of this complex was shown to be critical for the anti-apoptotic activity of Op-IAP3 [32]. Mammalian cellular IAPs (cIAPs) were first identified as proteins which bound indirectly to TNFR2 through TRAF1 and TRAF2 (see [33] for review). These proteins shared similarity in multiple BIR (Baculoviral IAP repeat) motifs and RING fingers, although the functions of these domains were unknown at that time. It was later established that IAPs bind to TRAFs (Figure 1) via their BIR1 domains [34,35]. Structural analysis showed that a single IAP molecule binds to a TRAF2 trimer [36,37]. The cellular XIAP protein was shown to bind to and inhibit caspase 3 and caspase-mediated apoptosis [38,39]. The BIR2 domain was required to inhibit both caspase 3 and 7 [40]. The BIR2 domain is required to bind processed caspase 3 and the inhibitory domain shown to lie between BIR1 and BIR2. An important development was the demonstration that some cellular IAPs have ubiquitin ligase activity mediated through the RING finger domains. This ubiquitin ligase activity is required for the ubiquitinylation of the RIPK1 complex (see [33] for review). Additional roles for cIAPs were discovered in inhibiting the pro-inflammatory cell death pathways, necroptosis and inflammasome activation leading to the pyroptosis cell death pathway. Consequences of inflammasome activation include the induction of caspase 1- and 11-driven lytic inflammatory cell death by pyropotosis or activation of inflammation mediated by cleavage of IL-1β precursor. Well beyond the inhibition of cell death pathways, XIAP proteins also have roles in regulating signalling from innate immune receptors that depend on their ubiquitin ligase function. The domain structure of BIR motif-containing proteins is very varied as described in the Interpro databases (available online: http://www.ebi.ac.uk/interpro/entry/IPR001370).

#### 3.2.2. The ASFV IAP Protein A224L

**Figure 3:**
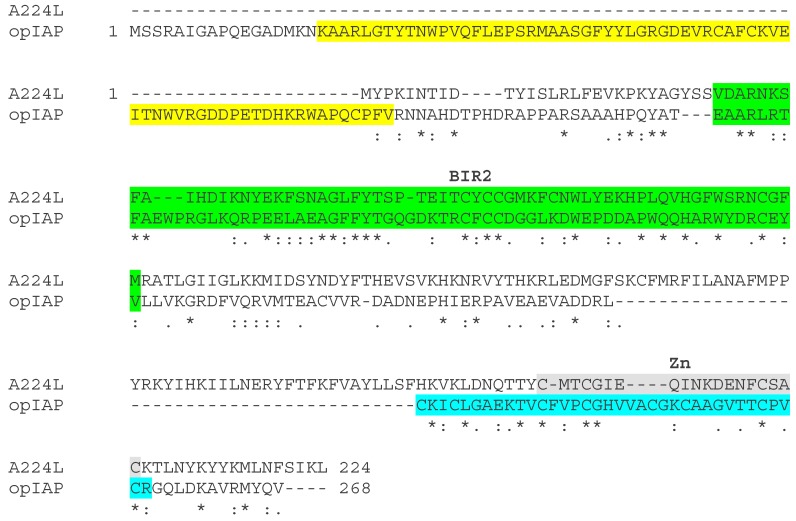
Comparison of ASFV A224L and opIAP protein sequences. The sequences of the ASFV BA71V isolate A224L protein (AOAOCAZXO) and Baculovirus opIAP (P41437) were aligned using Clustal Omega. The positions of domains in the proteins are indicated as coloured backgrounds. The BIR1 repeat (yellow) is present only in the opIAP protein. The BIR2 repeat is in both A224L and OpIAP sequences. At the C-terminus, A224L has a predicted C4 Zn binding domain (grey). The OpIAP protein contains a RING finger domain (turquoise). Identical amino acids are shown as asterisks (*) and similarities as double (:) or single (.) dots.

Studies on the function of the ASFV IAP-like protein, A224Lwere reported prior to 2002 lacking knowledge about the more recent evidence describing the role of IAPs in different cell death pathways and signalling. Consequently, information is lacking on the potential broader roles of A224L. The A224L protein is 224 amino acids long and well conserved in different ASFV isolates sharing 90% to 99% amino acid identity. Comparison with other members of the IAP family identified between 25% and 34% amino acid identity compared to Baculovirus IAP family proteins between region 17 to 97 amino acids (see Figure 3). The highest amino acid identity with cellular proteins is 32% with Drosophila between residues 19 to 97. This region encodes the A224L protein BIR repeat, which is between residues 29 to 92. A canonical RING motif is not present in A224L protein. The RING motif is present in many other IAP proteins and is required for the ubiquitin ligase activity, suggesting that A224L lacks this function. A224L contains a predicted zinc finger of the 4 cysteine type near the C-terminus (residues 189 to 207) in contrast to ring fingers of the C3HC4 type found in some other IAP (see Figure 3). Stable expression of the A224L IAP-like protein in cells substantially inhibited caspase 3 activity (Figure 1) and cell death induced by treatment with tumour necrosis factor α [41]. When transiently overexpressed, A224L inhibited cell death induced by cycloheximide or staurosporine. Proteolytic cleavage of caspase 3 was increased at later times in Vero cells infected with an A224L deletion mutant compared to wild-type virus. Thus, the A224L protein was indicated to promote cell survival, although the yield of infectious progeny virus was not affected by deletion of A224L. The data suggested that A224L may directly interact with the processed fragment of caspase 3 [41]. Expression of the A224L protein was detected late during infection and the protein was incorporated into virus particles, supporting a role for the protein late in infection or early after entry of the virus particle [42]. A possible alternative mechanism by which A224L may inhibit cell death was suggested from studies which showed that transient expression of A224L activated an NF-kB-dependent reporter. In cell lines stably expressing A224L PMA and ionomycin, stimulation induced greater levels of *c-rel*, an NF-kB dependent gene, than in control cells. This NF-kB inducing activity was abrogated by an IKK-2-dominant negative mutant and enhanced by expression of TNF receptor-associated factor 2 [43]. The activation of NF-kB mediated by TNF-R2 can inhibit apoptotic cell death by activating transcription of a number of anti-apoptotic genes including *IAP* and *Bcl-2* family members. Activation of NF-kB also drives expression of *cFLIP*, an inactive caspase 8 homolog that inhibits its activity (see [33] for review). Deletion of the A224L (*4CL*) gene from the virulent Malawi isolate did not affect levels of virus replication in porcine macrophage cell cultures or the infected macrophage survival time and the induction and magnitude of apoptosis. Moreover, the deletion of this gene from this virulent isolate did not reduce virulence in infected pigs [18]. Thus, although this A224L IAP-like protein was shown to be functional in mammalian cells in inhibiting cell death, no obvious phenotype associated with deletion of the gene was identified either during virus replication in macrophages or infection of pigs [18]. It is possible that the loss of the gene is compensated for by other cell-death inhibitors encoded by the virus. Given the more recently discovered roles of IAP proteins in other cell-death pathways and in cell signalling and inflammation, further investigation should be undertaken of the role of A224L protein in regulating these processes.

### 3.3. ASFV Inhibition of the Stress-Activated Apoptosis Pathway

The pro-apoptotic CCAAT-enhancer-binding protein homologous protein (CHOP) pathway is a cell-death pathway that is activated in cells in response to stress signals including virus infection and the unfolded protein response. This pathway can be induced following phosphorylation of the translation initiation factor eIF2-α by a family of stress-activated protein kinases. These include the double-stranded RNA-activated protein kinase, PKR, which is activated following infection with many viruses including poxvirus but not ASFV. The endoplasmic reticulum resident protein kinase, PERK, is activated during the unfolded protein response (Figure 1). Phosphorylation of eIF2-α on serine 51 leads to reduction of global protein synthesis due to the increased affinity of eIF2-α for the guanine nucleotide exchange factor, eIF2B, which limits formation of the pre-initiation complex required for protein translation. A small subset of proteins can still be translated when eIF2-α is phosphorylated. These include the transcription factor ATF4 and downstream targets including the pro-apoptotic transcription factor CHOP [44,45]. CHOP decreases transcription of Bcl2, depletes cellular glutathione and increases production of reactive oxygen species, sensitising the cell to ER stress and apoptosis [46]. CHOP participates in oxidative stress-mediated apoptosis though the induction of ER oxidase 1α (ERO1α), hyperoxidising the lumen which may result in leakage of hydrogen peroxide into the cytoplasm [47]. 

**Figure 4:**
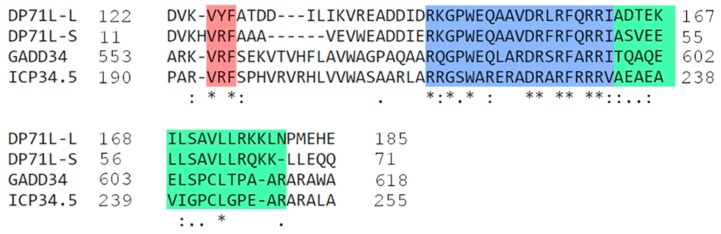
Alignment of ASFV DP71L with GADD34 and ICP34.5 of HSV-1. The long and short forms of DP71L share significant homology with the C-terminal domain of ICP34.5 of HSV-1 and GADD34. Within the C-terminal region of ICP34.5, residues 233–248 (shaded green) have been identified as the eIF2α binding domain [55]. The LSAVL motif within this was identified as critical for function [48]. The eIF2α binding motif described [56] in GADD34 is highlighted in blue. Identical amino acid residues shard between the sequences are shown with an asterisk (*) and similarities are shown as double (:) or single (.) dots.

The ASFV DP71L protein shares similarity in a C-terminal domain with the HSV ICP34.5 protein and the host GADD34 and CreP proteins. This protein is encoded by most analysed ASFV isolates as a short form of 71 or 72 amino acids (DP71Ls). In some isolates (for example, genotype VIII from Malawi), a longer form of the protein with an amino-terminal extension of about 112 amino acids (DP71Ll) is encoded. The DP71L protein is expressed late during the replication cycle. The DP71Ls proteins share 94% to 100% amino acid identity with each other. The DP71Ll proteins share 61% identity with DP71Ls over the domains 7 to 71 in DP71Ls and 118 to 185 in DP71Ll (see Figure 4). Amino acid identities of DP71Ls over this domain are approximately 30% to 40% with ICP34.5, GADD34 and CreP proteins; All of these proteins recruit protein phosphatase 1 to dephosphorylate eIF-2 α and restore global protein synthesis [45,48,49,50,51,52] (Figure 1). ASFV activates PP1 and promotes the expression of GADD34 [9]. As a consequence, exogenously expressed DP71L protein can inhibit the stress-induced induction and activation of the pro-apoptotic CHOP protein. Induction of CHOP is inhibited in ASFV infected cells, even those infected with ASFV lacking the *DP71L* gene. This suggests that ASFV may encode other inhibitors of this pathway [50]. The deletion of the DP71L protein from one isolate (E70) reduced virus virulence in pigs, whereas from another isolate (Malawi LIL20/1) no reduction was observed [53,54]. It is possible that the different virus gene complements may explain these differences in results. 

### 3.4. The ASFV C-Type Lectin Domain Containing Protein

The ASFV protein, EP153R, was indicated to inhibit the induction of apoptosis. Increased caspase 3 activity and cell death were observed in cells infected with an *EP153R* gene-deletion mutant as compared with infection with the parental BA71V strain. Both transient and stable expression of the *EP153R* gene in cells resulted in a partial protection of the cells from apoptosis induced in response to virus infection or external stimuli. EP153R reduced the transactivating activity of the cellular protein p53 following induction of apoptosis (Figure 1). Since p53 activates transcription of a number of apoptosis inhibitors, this could explain the mechanism of EP153R activation [57,58]. 

## 4. Role of Apoptosis in Pathology and Immune Responses

### Apoptosis in Tissue Samples from ASFV Infected Pigs

In lethal forms of ASF, early leukopenia is frequently described due to a decrease in the number of circulating monocytes, B- and T- lymphocytes [59,60]. In addition, pronounced depletion of lymphoid tissues is a hallmark of ASF that has been associated with lymphopenia. Other changes observed include severe vascular lesions affecting different organs and body cavities such as hyperemic splenomegaly, haemorrhages or edemas, thrombocytopenia and the induction of disseminated intravascular coagulation. Here, we summarize studies on apoptosis in lymphoid and non-lymphoid organs of pigs infected with ASFV isolates.

**Figure 5:**
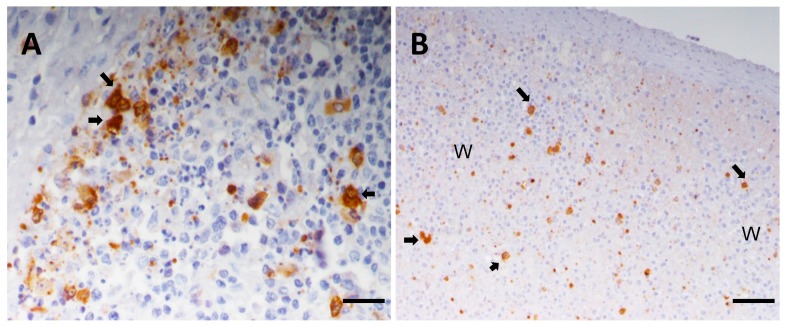
Immunohistochemical detection of ASFV protein P30 on wax-embedded tissue sections from pigs inoculated with the highly virulent ASFV isolate OURT88/1 and euthanized at day 5 post-infection. (**A**) Tonsil, Bar 40 μm. Lymphoid follicle with severe lymphoid depletion. Observe the presence of infected macrophages (arrows) close to areas where lymphocytes show characteristic features of apoptosis such as reduced size and hyperchromatic nuclei. Cell debris and apoptotic bodies, many of them immunolabeled, are also observed; (**B**) spleen, Bar 80 μm. Note the presence of infected cells, mainly macrophages (arrows), along with pyknotic cells, cell debris and apoptotic bodies immunolabeled against P30 in white pulp areas (WP) with severe lymphoid depletion.

ASFV replicates in cells of the mononuclear phagocyte system, predominantly monocytes and fixed-tissue macrophages [8,61,62]. However several other cell types were shown to be infected, especially in the later stages of the disease [60,63]. Infection induces apoptosis of infected macrophages in vivo and the massive apoptosis of bystander lymphocytes (Figure 5) is one of the hallmarks of the acute disease [8,61,62]. Widespread cell death was observed in infected cells of the mononuclear phagocyte system due to programmed cell death. However, at later stages of the disease, cytophatic effects with morphological changes of necrosis are identified in ultrastructural studies [61,62,63,64,65].

Both lymphoid depletion in primary and secondary lymphoid organs and the death of infiltrate associated with the lymphocytes in non-lymphoid organs such as liver and kidney have been attributed to massive apoptosis of lymphocyte subsets [8,61,62]. The mechanisms by which this apoptosis is induced are poorly understood. Lymphocyte destruction was observed mainly in T areas of retropharyngeal lymph nodes, gastrohepatic lymph nodes [64] and tonsils [65] from pigs infected with a highly virulent isolate. Many apoptotic lymphocytes and apoptotic bodies appeared to be phagocytosed by macrophages. There is no reported evidence for ASFV virus replication in cells of lymphoid origin.

The massive lymphocyte apoptosis that affected both B and T areas of lymphoid tissues was not infection of these cells by ASFV. However, the presence of infected macrophages close to areas with intense apoptotic phenomena suggested that the infected cells may have an indirect effect on uninfected lymphocytes such as secretion of chemical mediators by these macrophages [61,64].

A comparison of infections with a virulent isolate in susceptible domestic pigs or bushpigs, which do not develop clinical signs of acute ASF suggested lower virus replication in bushpigs was correlated with reduced secretion of cytokines or vasoactive substances, and less lymphocyte apoptosis [66]. This could in part explain why bushpigs survive infections with virulent isolates of ASF. Further research is required to support this tentative hypothesis.

Gómez del Moral et al. [67] demonstrated that TNFα containing supernatants from macrophage cultures infected with the ASFV virulent isolate Spain-75 induced apoptosis in uninfected lymphocytes. This effect was partially abrogated by pre-incubation with the anti-TNFα specific antibody. TNFα transcripts were detectable at 2–3 dpi in the liver, spleen and lymph nodes and correlated with viral protein expression. Elevated TNFα concentrations in serum were correlated to the onset of clinical signs in pigs. This confirmed a role for TNFα and probably additional pro-apoptotic factors in induction of lymphocyte apoptosis. TNFα-producing cells and infected cells were both identified as macrophages by immunohistochemistry in frozen samples of spleen and lymph nodes from infected pigs but not in non-infected controls [67].

Systematic analysis of pigs infected with the highly virulent isolate Spain-70 showed an increase in serum levels of TNFα and IL-1β from day 2 post-infection (dpi) [68]. From 3 dpi, pigs also displayed a severe leukopenia due to a decrease of circulating lymphocytes and monocytes [69]. Lymphoid depletion that affected both B and T areas was evident from 3 dpi (spleen, lymph nodes, tonsils), 4 dpi (thymus) and correlated with the presence of massive apoptotic phenomena. [68,69,70,71]. Infected cells, mainly macrophages, were detected close to apoptotic areas from 1 dpi in the spleen and lymph nodes and from 3 dpi in the thymus and tonsils. Immunohistochemistry showed significant increase of macrophages secreting cytokines. TNFα was secreted at a higher and more constant rate, while secretion of IL-1α was only detected at an early stage. A similar sequence was observed in the liver, where apoptosis in cell infiltrates was described from 5 dpi [72].

In summary, apoptosis affected both macrophage target cells and uninfected lymphocytes from initial stages of disease. An increase of macrophage counts in different areas of lymphoid organs was observed that coincided with the appearance of infected cells, mainly macrophages, and preceded massive lymphocyte apoptosis and lymphoid depletion typically associated with lethal forms of ASF. Macrophage activation was associated with the release of cytokines, mainly TNFα and IL-1α capable of inducing lymphoid tissue destruction by apoptosis. So, the presence of the virus might induce an increase of cytokine secretion in non-infected adjacent cells as a result of an autocrine effect. Lymphocyte apoptosis was correlated with the penetration of ASFV into organs and structures. Controlled apoptosis in lymphocytes would result in a diminished immune response enabling ASFV-infected cells to evade the immune system and replicate.

## 5. Conclusions and Future Work

As with other viruses, host cells respond to ASFV infection by initiation of apoptosis to limit virus replication. ASFV infection is sensed at the stage of virus entry before the onset of viral protein synthesis to activate caspase 3 and initiate apoptosis. Also, caspase 9 and 12 play an important role in apoptosis induction both by the mitochondrial pathway and the extrinsic pathway of ER stress. However, several viruses-encoded proteins block induction of apoptosis to enable replication of progeny virions. The induction of apoptosis observed at late stages of ASFV infection would favour “silent” virus spread, avoiding the activation of inflammatory responses that are induced by other cell-death pathways including necroptosis and pyroptosis. The activation of inflammatory responses could result in virus clearance by cells of the innate response, limiting virus replication.

ASFV encodes two anti-apoptotic proteins with similarity to cellular protein families. The A179L Bcl-2 like protein has an unusually broad specificity of binding to pro-apoptotic BH3-domain containing proteins. A179L is presumed to function by neutralising the pro-apoptotic function of these cellular proteins. The preferred binding partners for A179L have been indicated by direct binding and interactions in uninfected cells. The binding partners of A179L in infected cells and the impact of expression on infection in cells and in animals remain to be confirmed. Evaluating the role of A179L in virus persistence in wild suids in Africa and soft tick vectors would be of great interest.

The ASFV A224L IAP-like protein acts to inhibit apoptosis by two mechanisms, direct binding and inhibition of caspase 3 and activation of the NF-kB transcription factor and of the anti-apoptotic genes it controls. A224L lacks the RING finger domain that is present in some cellular IAP proteins, suggesting that it lacks ubiquitin ligase function, but this has yet to be confirmed. Cellular IAP proteins have a role in inhibition of necroptosis and pyroptosis and signalling. Potential functions of A224L in these pathways should also be investigated. Additional modulators of cell-death pathways in infected cells undoubtedly remain to be identified. In this context, it will also be interesting to investigate the role of non-coding RNAs in regulating apoptosis during ASFV infection. For example, micro RNAs have been shown to negatively regulate mRNAs for proteins in a number of cellular pathways including apoptosis.

Mechanisms leading to the massive apoptosis of non-infected lymphocytes in lymphoid and non-lymphoid tissues observed in acute ASFV infections are poorly understood. The evidence indicates that factors secreted from ASFV-infected macrophages are involved and TNF-α is suggested to be at least one mediator of this process. Further investigation is needed to establish the key mediators of lymphocyte apoptosis in tissues. The lymphopenia observed in blood during acute ASFV infection is likewise poorly understood. This may involve, in addition to direct effects of ASFV-infected cells, indirect effects which influence homeostasis of lymphocyte populations.

A better understanding of the factors influencing cell death during ASFV infection will contribute to the understanding of disease pathogenesis and the development of effective vaccine strategies.
